# A mixed-methods evaluation of organization and individual factors influencing provider intentions to use caregiver coaching in community-based early intervention

**DOI:** 10.1186/s43058-024-00552-5

**Published:** 2024-02-27

**Authors:** Melanie Pellecchia, David S. Mandell, Liza Tomczuk, Steven C. Marcus, Rebecca Stewart, Aubyn C. Stahmer, Rinad S. Beidas, Sarah R. Rieth, Gwendolyn M. Lawson

**Affiliations:** 1 Center for Mental Health, Psychiatry Department, University of Pennsylvania, Perelman School of Medicine, Philadelphia, USA; 2 School of Social Policy and Practice, University of Pennsylvania, Philadelphia, USA; 3 University of California, Davis, Mind Institute, Sacramento, USA; 4 Department of Medical Social Sciences, Feinberg School of Medicine, Northwestern University, Evanston, USA; 5 College of Education, San Diego State University, San Diego, USA; 6 Child and Adolescent Services Research Center, San Diego, USA; 7 Department of Child and Adolescent Psychiatry and Behavioral Sciences, Children’s Hospital of Philadelphia, Philadelphia, USA; 8 Department of Psychiatry, University of Pennsylvania, Perelman School of Medicine, Philadelphia, USA

**Keywords:** Organizational culture, Organizational climate, Intentions, Multilevel implementation strategies

## Abstract

**Background:**

Most psycho-social interventions contain multiple components. Practitioners often vary in their implementation of different intervention components. Caregiver coaching is a multicomponent intervention for young autistic children that is highly effective but poorly implemented in community-based early intervention (EI). Previous research has shown that EI providers’ intentions, and the determinants of their intentions, to implement caregiver coaching vary across components. Organizational culture and climate likely influence these psychological determinants of intention by affecting beliefs that underlie attitudes, norms, and self-efficacy to implement an intervention. Research in this area is limited, which limits the development of theoretically driven, multilevel implementation strategies to support multi-component interventions. This mixed methods study evaluated the relationships among organizational leadership, culture and climate, attitudes, norms, self-efficacy, and EI providers’ intentions to implement the components of caregiver coaching.

**Methods:**

We surveyed 264 EI providers from 37 agencies regarding their intentions and determinants of intentions to use caregiver coaching. We also asked questions about the organizational culture, climate, and leadership in their agencies related to caregiver coaching. We used multilevel structural equation models to estimate associations among intentions, psychological determinants of intentions (attitudes, descriptive norms, injunctive norms, and self-efficacy), and organizational factors (implementation climate and leadership). We conducted qualitative interviews with 36 providers, stratified by strength of intentions to use coaching. We used mixed-methods analysis to gain an in-depth understanding of the organization and individual-level factors.

**Results:**

The associations among intentions, psychological determinants of intentions, and organizational factors varied across core components of caregiver coaching. Qualitative interviews elucidated how providers describe the importance of each component. For example, providers’ attitudes toward coaching caregivers and their perceptions of caregivers’ expectations for service were particularly salient themes related to their use of caregiver coaching.

**Conclusion:**

Results highlight the importance of multi-level strategies that strategically target individual intervention components as well as organization-level and individual-level constructs. This approach holds promise for improving the implementation of complex, multicomponent, psychosocial interventions in community-based service systems.

**Supplementary Information:**

The online version contains supplementary material available at 10.1186/s43058-024-00552-5.

Contributions to the literature

- Most psycho-social interventions comprise multicomponent intervention packages. Previous research has demonstrated variability in implementation across the components of complex interventions, but implementation strategies often do not account for barriers to using specific components.
- The current study advances implementation science by examining the association among theory-driven organization-level and individual-level constructs as they relate to the components of a multi-component intervention. Results highlight the promise of tailored, multi-level implementation strategies that strategically target both organization-level and individual-level constructs.
- These findings help fill gaps in the implementation science literature and offer guidance for improving the implementation of complex multicomponent interventions. The results suggest that it is important to develop implementation strategies that target the individual components of a complex psychosocial intervention, rather than the whole intervention package, to improve its implementation.

## Background

Most psychosocial interventions are composed of at least several components. Practitioners often vary in the extent to which they implement these different components [1]. Psychological determinants of implementation may vary across intervention components as well. For example, providers’ intentions to implement the core components of multicomponent interventions, and the determinants of their intentions, can vary by core component [2–4]. Organizational culture and climate likely interact with psychological determinants of intention by affecting beliefs that underlie attitudes, norms, and self-efficacy to implement an intervention. However, research in this area is limited, which limits the development of theoretically driven, multilevel implementation strategies to support multi-component interventions.

Caregiver coaching is a multicomponent, evidence-based intervention that is considered best practice for young autistic children, but is poorly implemented in community-based early intervention [5, 6]. Caregiver coaching includes a core set of components that should be implemented consistently during intervention sessions. The five core components are collaboration (partnering with the caregiver to make treatment decisions), authentic learning experiences (practicing strategies in real-life daily routines), demonstration (modeling strategies), practice and feedback (providing the caregiver with an opportunity to practice strategies and delivering in-vivo feedback), and reflection and problem solving (supporting the caregiver in reflecting on strategy use and problem-solving around any barriers) [7, 8].

Over the last decade, early intervention (EI) research for young autistic children has emphasized the importance of including caregivers as partners in intervention delivery via caregiver-mediated intervention [9, 10]. Several randomized trials, most relying on university-based clinicians to coach caregivers of young autistic children to deliver interventions to their child, find that caregiver-mediated early intervention results in significant improvements in children’s cognitive ability, social functioning, behavior, academic skills, and daily living skills [11–13] and leads to improved parental self-efficacy and engagement [14, 15]. In contrast to university-based studies of caregiver-mediated interventions, outcomes of community-based EI for young autistic children tend to be poor, especially in low-income and minority communities [16–18]. These poor outcomes may be due in part to a lack of effective caregiver coaching. EI practitioners rarely coach caregivers of young autistic children [6, 19]. Instead, they spend most of their time working directly with the child [20, 21]. This is especially true for families from traditionally marginalized or minoritized backgrounds. EI practitioners have reported challenges with coaching and a preference for intervening directly with the child when working with caregivers from marginalized backgrounds [22]. A recent study found that EI practitioners implement caregiver coaching based on perceptions of caregiver “readiness” for coaching, which depended on factors such as caregiver session attendance, caregivers directly asking for advice, and caregiver attentiveness during sessions [23]. These findings highlight factors that may lead to disparities in access to caregiver coaching, especially for those who may benefit the most, and point to the critical need for an in-depth understanding of the barriers to implementing caregiver coaching in publicly funded EI systems.

**Fig. 1:**
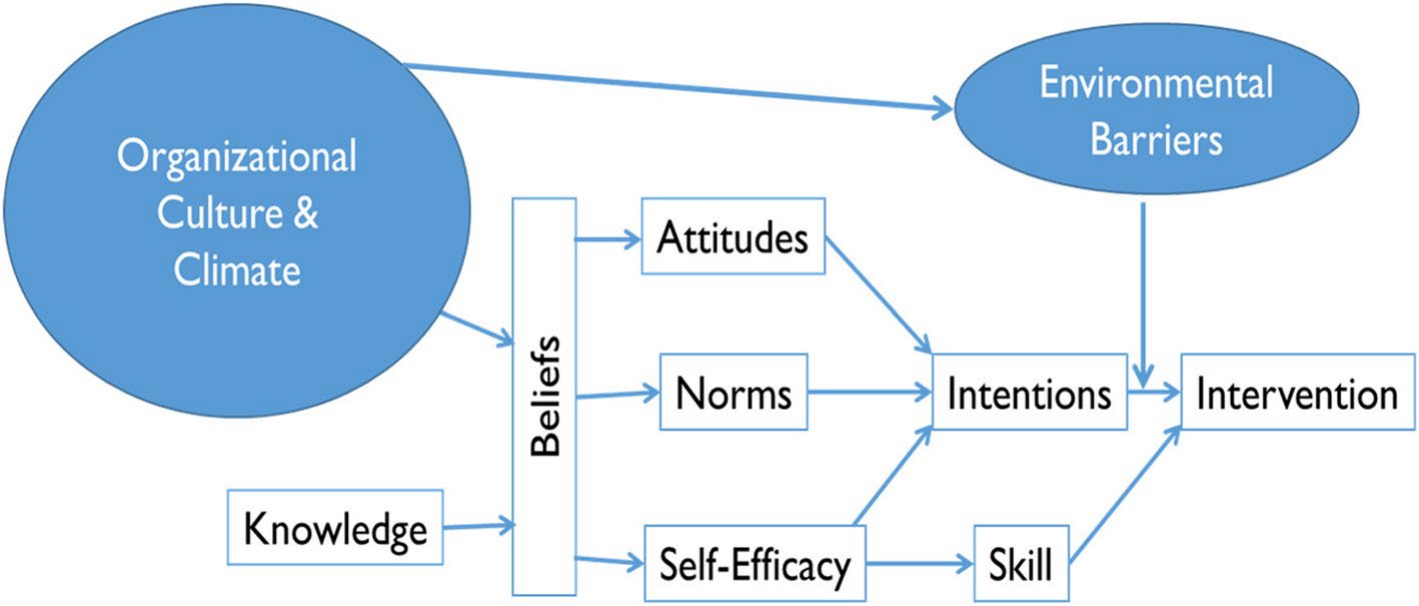
Conceptual model of factors affecting implementation

Recent studies designed to develop and test causal models and mechanisms in implementation research highlight the importance of both individual and organizational factors as potential implementation levers [24, 25]. Tailored implementation strategies that target specific mechanisms associated with the use of discrete components of multicomponent interventions may be more successful than broad implementation strategies in changing practitioner behavior. A recent theory of change highlights the importance of integrating both psychological and organizational theories of behavior change into implementation research when evaluating causal pathways (see Fig. 1) [26]. Psychological theories of behavior change can provide insight into why EI practitioners do or do not use the components of caregiver coaching and inform strategies to improve its implementation. Leading behavior change theories posit that an individuals’ intention to perform a certain behavior is the most proximal determinant of that behavior when individuals have the ability to act on their intentions [27]. Intentions represent an individual’s motivation to perform a certain behavior and are malleable targets for implementation research [26]. The determinants of intention are attitudes (e.g., whether the practitioner “likes” or “dislikes” coaching caregivers), descriptive norms (e.g., whether the practitioner perceives that other practitioners like them use coaching), injunctive norms (e.g., whether the practitioner perceives that important others, such as supervisors, expect them to use it), and self-efficacy (e.g., whether the practitioner believes that they have the necessary skills to provide effective coaching). This model has been used to predict health behaviors [28] and to predict the use of evidence-based practices in schools [29, 30], including those specific to autism [4]. Meta-analytic reviews that evaluate the association between intentions and behavior find that intentions are most likely to predict behavior if the individual has the skills and resources needed to perform the behavior [31, 32]. These reviews find that perceived behavioral control accounted for significant amounts of variance in intention and behavior, independent of other constructs. Furthermore, intentions were better predictors of behavior, than attitudes, subjective norms and perceived behavioral control [32]. Therefore, evaluating an individual’s intentions to perform a certain behavior or use a specific practice facilitates the study of mechanisms that influence both intention formation and factors that facilitate or impede an individual from acting on their intention to use a practice [26, 33]. Previous work finds substantial variability in practitioners’ intentions to implement new evidence-based practices (EBPs) and that those intentions are associated with subsequent implementation [4]. A recent study examining EI providers’ intentions to use the core components of caregiver coaching found that intentions varied by core component, and the associations between attitudes, norms, self-efficacy, and intentions also varied by caregiver coaching component. For example, EI providers self-efficacy predicted their intention to use collaboration with parents and deliver the intervention within daily routines, but not to other components of caregiver coaching [2].

The theory of change shown in Fig. 1 also highlights the potential importance of organizational factors in affecting implementation. Specifically, organizational-level factors (such as climate, culture, and leadership) may influence individual-level factors (such as attitudes, norms, and self-efficacy) by affecting the beliefs that underlie these factors or may interact with these individual-level factors to affect implementation [26]. However, little work to date has examined these hypothesized relationships empirically.

Dimensions of organizational culture, climate, and leadership include both strategic (i.e., focused on the strategic goals of the organization, including EBP implementation) and global factors. Evidence suggests that both global and strategic organizational-level factors may be important for implementation. For example, global organizational culture and climate have been shown to predict attitudes that support EBP use [34]. Glisson and colleagues [35] define organizational culture as the shared norms and behavioral expectations that guide how work is prioritized and completed in the organization. Organizational climate represents staff perceptions of the impact of the work environment on the individual [36]. Strategic organizational-level factors include implementation leadership (i.e., leaders’ support for and perseverance during EBP implementation [37]) and implementation climate (i.e., employees’ shared perceptions of the importance of EBP implementation within the organization) [38]. Both implementation leadership and climate have been shown to predict evidence-based practice implementation [39]. Furthermore, previous research has shown that organizational culture influences employees’ reactions to changes, such as the introduction of a new educational or clinical practice. For example, public school teachers’ ratings of organizational culture as supportive were positively correlated with their intentions to implement new practices; these effects were mediated by teachers’ attitudes, norms, and self-efficacy regarding the new practices [40]. While this study gave some preliminary evidence of the relationships between organizational and psychological characteristics in affecting implementation, research in this area is limited, which limits the development of theoretically driven, multilevel implementation strategies.

Examining relationships among organizational factors and psychological factors affecting caregiver coaching implementation can inform strategies to improve use of multi-component interventions. There are many paths by which organizational culture and climate could affect EI providers’ intentions to coach caregivers. For example, providers in agencies that provide limited training and supervision in caregiver coaching may have weak self-efficacy in coaching caregivers, which may lead to weak intentions to use this approach. Conversely, providers in agencies with strong leadership support for caregiver coaching may have strong perceived descriptive and injunctive norms regarding caregiver coaching, which may lead to strong intentions. A better understanding of the relationship among these organizational-level and individual-level factors would provide critical insights needed to develop targeted, multilevel implementation strategies supporting the implementation of caregiver coaching for families of young autistic children receiving community-based early intervention.

The goal of this mixed methods study was to elucidate relationships among EI providers’ intentions to implement the components of caregiver coaching, psychological determinants of intentions, and organizational leadership, culture, and climate. We used the conceptual model presented in Fig. 1 as a backdrop to systematically test the relationships between organizational culture and climate, psychological determinants of intentions, and intentions to implement caregiver coaching in publicly funded early intervention. Because strategic organizational factors (i.e., implementation climate and leadership for caregiver coaching) are most proximal to caregiver coaching, we hypothesized about the association between these factors and intentions and their determinants. Specifically, we hypothesized that supportive implementation climate and leadership would be associated with favorable attitudes, norms, and self-efficacy for caregiver coaching among EI providers, which would in turn be associated with stronger intentions for caregiver coaching. We also examined the same relationships with global organizational culture and climate, although we did not have a priori hypotheses about these relationships. We used quantitative methods to examine the associations among these constructs (i.e., testing paths between hypothesized organizational-level constructs and EI providers’ attitudes, norms, and self-efficacy; and between attitudes, norms, and self-efficacy and intentions) and used qualitative methods to expand on the quantitative results.

## Method

### Procedures

Data for this study were collected to examine the constructs and theory of change described above as they relate to the use of caregiver coaching in publicly funded early intervention service systems.

### Mixed-methods

We relied on the established taxonomy of mixed-methods designs outlined by Palinkas and colleagues to guide our mixed-methods data collection and analysis [41]. We employed a sequential structure, beginning with the quantitative data, for the primary purpose of confirmation and hypothesis testing (i.e., QUAN > qual). The function of the qualitative component was to expand on and explain the quantitative findings. Our analyses connected the qualitative findings to the quantitative findings to gain a more nuanced and in-depth understanding of the organizational and individual-level constructs of interest.

### Quantitative methods

#### Participants

Agency recruitment occurred through the study team and their academic colleagues who are engaged in community-based early intervention autism research. Partnering agencies and system administrators shared information about the study to their networks of early intervention agencies. We attended staff meetings in-person at local agencies to share information about the study. Providers were eligible to complete the surveys if they (1) were employed by an EI agency in any professional discipline and (2) currently served at least three children classified as at high likelihood for autism spectrum disorder (ASD) on their caseload. Children under 3 years of age do not need a medical diagnosis of ASD to be eligible for publicly funded autism-related services. They are eligible if they are identified as at high likelihood for ASD.

Children with identified or suspected disabilities under 3 years of age in the USA are entitled to free or low-cost early intervention services under Part C of the Individuals with Disabilities Education Improvement Act. The treatment philosophy for Part C early intervention is focused on family-centered care, especially in supporting and empowering caregivers to support their children’s development. There has been increased emphasis on using caregiver coaching in Part C EI systems as a method to empower caregivers. We invited 358 EI providers (in person during staff meetings when possible or via survey link) from 44 agencies in Part C EI systems in southeastern Pennsylvania, southern California, Delaware, and Ohio to participate; 264 of these EI providers consented to complete the survey about EI providers’ demographics and professional background, their intentions to use core components of caregiver coaching, and measures of their organizational context (see Measures). The analytic sample consisted of 263 EI providers from 35 agencies who had available data on at least one measure of interest. The number of responding providers within each agency ranged from 3 to 44. Table 1 provides demographic characteristics of the quantitative sample. The sample was mostly White and female, and most participants had obtained a graduate/professional degree. Participants had an average of 7.7 years of experience working in early intervention (SD = 8.3). Most (67.7%) reported that they were independent contractors; 25.9% were full-time employees, and 7.6% were part-time employees. All providers reported having previous experience and training in caregiver coaching.

**Table 1:** Participant demographics and professional characteristics) for the quantitative (*N* = 263) and qualitative sample (*N* = 36)

Variables	*% of quantitative sample (N* = *263)*	*% of qualitative sample (N* = *36)*
Gender:		
Female	95.8%	100%
Male	4.2%	0%
Race:		
White	76.8%	66.7%
African-American or Black	8.4%	11.1%
Asian	8.4%	11.1%
American Indian/Alaska Native	1.5%	0%
Pacific Islander	.8%	0%
Middle Eastern	.8%	0%
Other	6.8%	0%
Ethnicity: Latino/Hispanic/Spanish	15.2%	11.1%
State:		
PA	70.7%	75.0%
CA	26.6%	19.4%
DE	2.3%	5.6%
OH	.4%	0%
Job title:		
Speech and language pathologist	23.6%	16.7%
Occupational therapist	17.5%	11.1%
Special instructor	27.4%	47.2%
ABA therapist	14.1%	0%
Physical therapist	10.3%	22.2%
Other	12.9%	5.6%
Employee type:		
Full-time employee	25.9%	24.3%
Part-time employee	7.6%	6.7%
Independent contractor	67.2%	56.8%
Other	1.1%	0%
Unknown	0%	12.2%
Highest level of education		
Some college	1.1%	0%
College	18.3%	19.0%
Graduate/professional degree	79.8%	81.0%
Other	.8%	0%

Due to missing data, the sample size for the quantitative sample was* N* = 262 for the gender and highest level of education variables

We administered the surveys in person at 3 agencies during staff meetings, from which we collected 37 surveys. At the other 41 agencies, agency leaders distributed information about the survey to all EI providers in their agency. Interested EI providers contacted the study team, which provided a secure, unique survey link via email. Participants who completed the survey received a $45 electronic gift card.

### Quantitative measures

#### Demographic and background characteristics

Participants completed a short questionnaire about their socio-demographic and professional background characteristics, including gender, race, ethnicity, job title, whether they were full time, part time or contracted employees, years’ experience working in early intervention, and specialized training.

#### Implementation Climate Scale (ICS)

The ICS is an organization-level measure of implementation climate. It measures six dimensions of implementation climate: focus on EBP, educational support for EBP, recognition for EBP, rewards for EBP, selection for EBP, and selection for openness [42]. It contains 18 items measured on a 5-point scale from 0 = “not at all” to 5 = “to a great extent.” We modified the ICS to probe about implementation climate related to caregiver coaching. In the current sample, the ICS had high internal consistency (*α* = 0.95) and an intraclass correlation coefficient (ICC) of 0.29, indicating that approximately 29% of the total variation in total ICS score could be accounted for by agency. The mean *r*_wg_ value among agencies with at least 10 respondents was 0.692. These values are consistent with observed ICC and *r*_wg_ values typically found in the literature for team-level constructs [43]. Consistent with our conceptualization of this construct as an agency-level construct, prior research (e.g., Williams et al., 2020), and these data, we aggregated the providers’ individual ICS scores as an agency-level variable. Because we were primarily interested in the role of overall implementation climate for caregiver coaching, and to reduce the number of models tested, we used the mean ICS score, aggregated at the agency level, in the analyses.

#### Implementation Leadership Scale (ILS)

The ILS is an organization-level measure of aspects of leadership that relate to implementation. This scale assesses the degree to which a leader is knowledgeable, supportive, proactive, and perseverant in implementing EBPs. It contains 12 items measured on a 5-point scale from 0 = “not at all” to 5 = “to a great extent.” We modified the item stems to probe about leadership support related to caregiver coaching, to improve the specificity of the responses [37]. In the current sample, the total ILS had high internal consistency (*α* = 0.97) and an intraclass correlation coefficient (ICC) of 0.20, indicating that approximately 20% of the total variation in total ILS score could be accounted for by agency. The mean *r*_wg_ value among agencies with at least 10 respondents was 0.689. Consistent with our conceptualization of this construct as an agency-level construct, prior research (e.g., Williams et al., 2020), and these data, we aggregated the providers’ individual ILS scores as an agency-level variable. Because we were primarily interested in the role of overall implementation leadership for caregiver coaching, and to reduce the number of models tested, we used the mean ILS score, aggregated at the agency level, in the analyses. Analyses with the ILS variable used data from 262 clinicians, who reported on their supervisors, that had available data on this measure.

#### Organizational Social Context (OSC)

The OSC measures the organizational culture and climate of social service organizations. It is a nationally normed and psychometrically validated 105-item scale that measures cultures and climates of direct service agencies [35]. The OSC provides scores on six subscales. Three subscales (proficiency, rigidity, resistance) measure culture. Proficient cultures are characterized by expectations that service providers will place the well-being of each client first and by expectations that individual service providers will be competent and have up-to-date knowledge. Rigid cultures are characterized by service providers having less discretion and flexibility in their work, limited input into key management decisions, and being controlled by many bureaucratic rules and regulations. Resistant cultures are characterized by expectations that service providers will show little interest in change or in new ways of providing service. The other three OSC subscales (Engagement, Functionality, Stress) measure climate. Engaged agencies are characterized by employee perceptions that they are able to personally accomplish many worthwhile things in their work. Functional climates are characterized by employee perceptions that they receive the cooperation and help from coworkers and administration required to do their job. Stressful climates are characterized by employee perceptions that they are emotionally exhausted from their work, pulled in different directions, and unable to get the necessary things done. We included the subscales separately in our analysis. The OSC scales demonstrated adequate reliabilities, with no scales falling below the typical 0.70 alpha cutoff. The intra-group agreement indices (*r*_wg_) were all above the suggested 0.70 level. Analyses with the OSC variable used data from 242 clinicians from 18 agencies that had available data on this measure.

#### Intentions

Participants were asked to rate their strength of intention to use each of the five core components of caregiver coaching: (1) collaboration, (2) practicing strategies in real-life daily routines, (3) demonstration, (4) practice and feedback, and (5) reflection and problem solving at almost every parent session. The items used validated stems [44] for each caregiver coaching component (e.g., “How likely are you to give immediate feedback to caregivers after they attempt a technique at almost every caregiver session?”). Each item was rated on a 7-point scale (1 = Extremely unlikely to 7 = Extremely likely) with higher numbers representing stronger intentions.

#### Psychological determinants of intentions

Participants reported their attitudes, descriptive norms, injunctive norms, and self-efficacy about each core component of caregiver coaching using established question stems for each domain [44]. Participants reported their attitudes about each core component of caregiver coaching (i.e., “Think about what it would be like if you almost always demonstrated intervention techniques to caregivers during sessions. Would that feel…”) using four items with bipolar anchors—inappropriate-appropriate; stressful-calm; inconvenient-convenient; useless-helpful—rated on a scale from 0 to 10 for each item. Participants also reported descriptive norms using two items (i.e., “At my organization, most providers will do this”; “Most providers who work with similar caregivers are willing to do this.”) on a 5-point scale from 1 = strongly disagree to 5 = strongly agree for each of the coaching components. Injunctive norms were measured using the question stem: “If you almost always collaboratively made decisions with caregivers during a session, how would the following groups of people feel about you doing that?”, with a 5-point scale ranging from 1 = strongly disapprove to 5 = strongly approve for two groups of people: “My boss/supervisor,” and “Colleagues who are important to me.” Finally, participants reported on self-efficacy for each core component of caregiver coaching using the item “I am confident that, if I wanted to, I could do this” rated on a 5-point scale ranging from 1 = strongly disagree to 5 = strongly agree. See [45] for a comprehensive description of the question stems used to assess these psychological determinants of intentions.

### Quantitative analyses

**Fig. 2:**
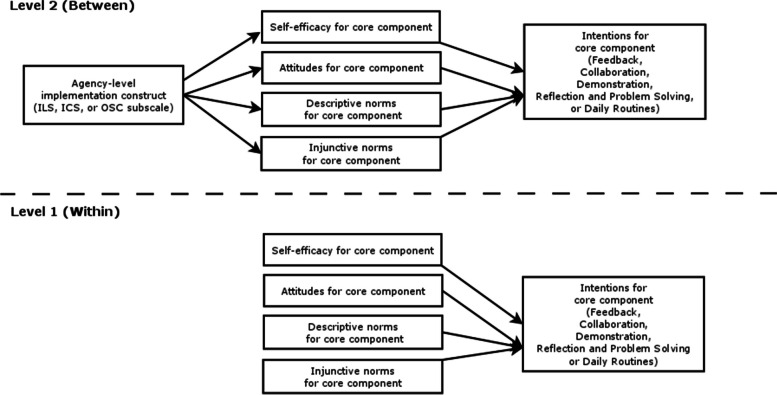
Path diagrams for the quantitative models. Separate models were tested for each agency-level implementation construct (i.e., ILS, ICS, or OSC subscale) and for each caregiver coaching core component (i.e., feedback, collaboration, demonstration, reflection and problem solving, daily routines)

We used MPlus Version 8.6 [46] to run multilevel path models, specifically 2–1-1 Multilevel Structural Equation Models [47], to estimate associations among organizational factors (i.e., ICS total score, ILS total score, OSC subscale scores), psychological determinants of intentions (i.e., attitudes, descriptive norms, injunctive norms, and self-efficacy), and intentions. At level 1 (within-agency), we specified paths from self-efficacy, attitudes, descriptive norms, injunctive norms, and self-efficacy to intentions to use the caregiver coaching core component. At level 2 (between-agency), we specified paths from the organizational construct of interest (i.e., ICS total score, ILS total score) to the psychological determinants of intentions and paths from each of the psychological determinants to intentions to use the caregiver coaching core component. We ran separate models to separately examine the separate contributions of the ICS and ILS on each caregiver coaching core component and to reduce concerns about potential multicollinearity. Path diagrams for the models are shown in Fig. 2.

### Qualitative methods

#### Qualitative study sample

We conducted semi-structured interviews with targeted subgroups of EI providers. Specifically, we recruited providers who reported high and low intentions to implement caregiver coaching through our quantitative surveys in order to gather rich perspectives from providers with the full range of intentions. High intentions were defined as a score of 6 or 7 on the intentions survey (7 = extremely likely to coach caregivers) while low intentions were defined as a score of 1 or 2 (1 = extremely unlikely to coach caregivers). We randomly selected providers who reported high and low intentions to coach caregivers during their usual EI sessions and invited them to participate in follow-up interviews. The qualitative sample was balanced across providers who reported high and low intentions to coach caregivers. Recruitment procedures and inclusion criteria for the interviews were identical to that of survey participation. We obtained informed consent prior to participation. Participants were compensated $25 for participating in the interview. All study procedures were approved by the University of Pennsylvania and City of Philadelphia institutional review boards. Of the 48 providers who were invited, none explicitly refused to participate, although 12 did not return emails requesting their participation. We reached thematic saturation after 36 interviews from providers at 25 agencies. No repeat interviews were conducted. Providers varied in their disciplinary backgrounds: 47.2% were special instructors (a job title referring to therapists or instructors with a background in psychology, early childhood education, or a related field who visit families’ homes to provide intervention), 22.2% were physical therapists, 16.7% were speech and language pathologists, 11.1% were occupational therapists, and 5.6% classified themselves as other types of therapists, such as dieticians or developmental specialists. Providers we interviewed had an average of 9 years of experience working in EI, ranging from 0.5 and 39 years.

### Interviews

Interviews occurred either in person in the community, at places like public libraries, or via video conference, and took 30 to 45 min. Only the members of the research team and the participant were present during the interviews. Participants were informed about the purpose of the research. A bachelor’s level research coordinator or a doctoral level research investigator conducted the interviews. Interview training for interviewers included participating in a multiday workshop in qualitative research methods and data analysis. We developed the interview guide iteratively under the guidance of an expert in qualitative research and feedback from a community advisory board using the Consolidated Framework for Implementation Research (CFIR) and the Theory of Planned Behavior [27, 48, 49]. The interview, which was pilot tested prior to implementation, queried about (1) caregiver coaching strategies that EI providers use during interactions with caregivers, (2) views about the acceptability and appropriateness of caregiver coaching in EI, and (3) contextual factors that may influence the coaching strategies EI providers use with families. We selected prompts to probe for barriers and facilitators at the intervention, provider, organization, and caregiver level. We also probed for information about the supports needed to implement coaching in daily practice.

### Mixed-methods data analysis

All interviews were audio recorded, transcribed, and imported into NVivo 12 software. Members of the study team developed a qualitative codebook through a collaborative and iterative process guided by the Consolidated Framework for Implementation Research [48] and consistent with an integrated inductive and deductive approach to develop an organized coding system that proceeded through several stages of data analysis [49–51]. First, the three coders independently read three interview transcripts and independently identified distinct themes that emerged from those transcripts. The coding team discussed and combined the list of themes through consensus discussion. Then, the coding team independently reviewed three additional transcripts and met again to adjudicate differences, develop coding rules, consolidate redundant concepts, and create additional codes to reflect new concepts not previously identified. The final codebook for the current analyses included codes that relate to the conceptual model shown in Fig. 1. Three members of the team coded interview text based on this codebook and then engaged in an iterative analysis process to connect the qualitative findings to the quantitative findings. Our goal was to gain a more nuanced and in-depth understanding of the organizational and individual-level constructs of interest and their relationships. We used the Consolidated Criteria for Reporting Qualitative Studies (COREQ) [52] reporting guidelines to report our qualitative methods and findings (see Supplemental materials).

## Results

### Quantitative findings

The mean agency-level ICS score was 1.89 (*N* = 35, *SD* = 0.70, range [0.19, 3.13]), and the mean agency-level ILS score was 2.50 (*N* = 35, *SD* = 0.80, range [0.33,3.83]). Descriptive statistics of OSC subscale scores are reported in the Supplemental materials (Supplemental Table 1). The agency-level ICS and ILS score correlated significantly with each other (*r* = 0.74, *p* < 0.001); each also correlated significantly with the OSC functionality organizational climate subscale (*r* = 0.77, *p* < 0.001 for ICS; *r* = 0.64, *p* < 0.001 for ILS) and OSC proficiency culture subscale (*r* = 0.61, *p* = 0.007 for ICS; *r* = 0.50, *p* = 0.03 for ILS) but not with the other OSC subscale scores.

Provider-reported intentions to use components of caregiver coaching varied by component. The mean score on the measure of intentions were: 5.86 (i.e., in between “Slightly Likely” and “Quite Likely;” *N* = 255, *SD* = 1.14; range [1, 7]) for feedback; 5.98 (i.e., in between “Slightly Likely” and “Quite Likely;” *N* = 253, *SD* = 1.19; range [1, 7]) for collaboration; 6.15 (i.e., in between “Quite Likely” and Extremely Likely;” *N* = 253, *SD* = 1.20; range [1, 7]) for demonstration; 5.93 (i.e., in between “Slightly Likely” and “Quite Likely;” *N* = 254, *SD* = 1.15; range [1, 7]) for reflection and problem solving; and 5.67 (i.e., in between “Slightly Likely” and “Quite Likely;” *N* = 255, *SD* = 1.31; range [1, 7]) for working within daily routines. See Lawson et al. [2] for descriptive statistics of attitudes, descriptive norms, injunctive norms, and self-efficacy.

Results of multilevel SEM models for the ILS are shown in Table 2. There were statistically significant paths from the ILS to injunctive norms for all five caregiver coaching core component models in the level 2 (between-agency) analyses (all unstandardized *b*s > 0.22, all *p*s < 0.05). Additionally, there were significant between-level paths from the ILS to self-efficacy for feedback (*b* = 0.24, *p* = 0.01) and demonstration (*b* = 0.23, *p* = 0.03) and from the ILS to descriptive norms for collaboration (*b* = 0.34, *p* = 0.01) and demonstration (*b* = 0.23, *p* < 0.05). In these models, there were significant paths at the within-agency level from attitudes to intentions for all five caregiver coaching core models (all *b*s > 0.21, all *p*s < 0.05) as well as from self-efficacy to intentions for collaboration (*b* = 0.31, p = 0.02) and daily routines (*b* = 0.54, *p* < 0.001); from descriptive norms to intentions for feedback (*b* = 0.19, *p* < 0.05) and reflection/problem solving (*b* = 0.29, *p* = 0.007); and from injunctive norms to intentions for collaboration (*b* = 0.32, *p* = 0.02), demonstration (*b* = 0.33, *p* = 0.01), and daily routines (*b* = 0.39, *p* < 0.05).

**Table 2:** Results from multilevel path models with paths from Implementation Leadership Scale (ILS) to psychological determinants of intentions and from psychological determinants to intentions for the five caregiver coaching core components (i.e., feedback, collaboration, demonstration, reflection/problem solving, daily routines)

	Caregiver coaching core component				
	Feedback	Collaboration	Demonstration	Reflection and problem solving	Daily routines
	Effect (*p*)	Effect (*p*)	Effect (*p*)	Effect (*p*)	Effect (*p*)
**Between-level**					
ILS →self-efficacy	**.24 (.01)**	.09 (.40)	**.23 (.03**)	.16 (.20)	.18 (.21)
ILS →attitudes	.58 (.06)	.33 (.14)	.38 (.11)	.24 (.21)	.51 (.06)
ILS → descriptive norms	.22 (.08)	**.34 (.01)**	**.23 (.047)**	.09 (.49)	.34 (.31)
ILS → injunctive norms	**.31 (.008)**	**.28 (.03)**	**.22 (.02)**	**.22 (.004)**	**.22 (.003)**
**Within-level**					
Self-efficacy → intentions	.12 (.14)	**.31 (.02)**	.07 (.53)	.06 (.60)	**.54 (< .001)**
Attitudes → intentions	**.29 (< .001)**	**.22 (.006)**	**.26 (< .001**)	**.38 (< .001)**	**.21 (.01)**
Descriptive norms → intentions	**.19 (.04)**	.14 (.11)	.14 (.12)	**.29 (.007)**	.14 (.57)
Injunctive norms → intentions	.10 (.51)	**.32 (.02)**	**.33 (.01)**	− .02 (.82)	**.39 (.048)**

Separate models were tested for each caregiver coaching core component (i.e., feedback, collaboration, demonstration, reflection and problem solving, daily routines). Unstandardized coefficients are shown

Results for the models with the ICS were similar (see Table 3). In the between-agency analyses, there were significant paths from the ICS to injunctive norms in the models for feedback (*b* = 0.34, *p* < 0.004), demonstration (*b* = 0.21, *p* < 0.003), and reflection and problem solving (*b* = 0.23, *p* = 0.02) as well as from the ICS to self-efficacy in models for feedback (*b* = 0.28, *p* < 0.001) and demonstration (*b* = 0.23, *p* = 0.001) and from the ICS to attitudes in models for feedback (*b* = 0.63, *p* = 0.03) and demonstration (*b* = 0.45, *p* = 0.02). Within-level results were similar as those with the models that included the ILS.

**Table 3:** Results from multilevel path models with paths from Implementation Climate Scale (ICS) to psychological determinants of intentions and from psychological determinants to intentions for the five caregiver coaching core components (i.e., feedback, collaboration, demonstration, reflection/problem solving, daily routines)

	Caregiver coaching core component				
	Feedback	Collaboration	Demonstration	Reflection and problem solving	Daily routines
	Effect (*p*)	Effect (*p*)	Effect (*p*)	Effect (*p*)	Effect (*p*)
**Between-level**					
ICS → self-efficacy	**.28 (< .001)**	.05 (.74)	**.23 (.001)**	.13 (.12)	**.18 (.04)**
ICS → attitudes	**.63 (.03)**	.20 (.39)	**.45 (.02)**	.22 (.36)	.57 (.50)
ICS → descriptive norms	.19 (.11)	.24 (.12)	.24 (.053)	.14 (.27)	.26 (.90)
ICS → injunctive norms	**.34 (.004)**	.23 (.08)	**.21 (.003**)	**.23 (.02**)	.31 (.78)
**Within-level**					
Self-efficacy → intentions	.13 (.13)	**.31 (.02)**	.07 (.45)	.05 (.51)	**.54 (< .001)**
Attitudes → intentions	**.30 (< .001)**	**.22 (.007)**	**.26 (< .001**)	**.37 (< .001)**	**.21 (.002)**
Descriptive norms → intentions	**.19 (.04)**	.14 (.24)	**.14 (.045)**	**.29 (.007)**	.13 (.09)
Injunctive norms → intentions	.10 (.44)	.**32 (.04)**	**.33 (.01**)	− .02 (.82)	**.39 (.006**)

Separate models were tested for each caregiver coaching core component (i.e., feedback, collaboration, demonstration, reflection, and problem solving, daily routines). Unstandardized coefficients are shown

For the models with each of the six subscales (i.e., three culture subscales and three climate subscale) of the Organizational Social Context (OSC) measure, there were not statistically significant paths from most OSC subscales to attitudes, self-efficacy, descriptive norms, or injunctive norms, although there were some exceptions (e.g., significant between-level paths from higher proficiency culture, engagement climate, and functionality climate to higher self-efficacy for some caregiver coaching core components). The full set of results with the OSC subscales are displayed in the supplemental materials (Supplemental Table 2).

### Mixed methods findings

Our qualitative findings provided a deeper analysis of how providers described the relationships between the key constructs in the conceptual model. We used the conceptual model presented in Fig. 1 to guide the interpretation and presentation of the mixed methods findings.

### Self-efficacy

The quantitative analyses found that self-efficacy predicted intentions to implement caregiver coaching for two of the core coaching components: collaborating with caregivers and working in daily routines. However, providers’ low self-efficacy and comfort using caregiver coaching was evident throughout the interviews. Providers often described their self-efficacy as a major factor that influenced their use of caregiver coaching. Even though the quantitative analyses did not find an association between providers’ self-efficacy and their intentions to use feedback with caregivers, providers often described low self-efficacy and discomfort delivering feedback to caregivers. For example, one provider stated: “Sometimes some parents, they like to say [to their child] like, ‘No, don’t do this” or they like to say ‘no’ a lot, but it’s uncomfortable to have to step in and say ‘Actually, if you tell them no, you’re not prompting them with the response.’” Similarly, providers often reported feeling low self-efficacy related to encouraging caregivers to practice intervention strategies during sessions, an integral part of delivering feedback. One provider shared an account of a case where she struggled with using caregiver coaching during her sessions with a particular family: “I think the piece that was the most difficult was the parent practice piece. I mean she’d stick around for a demonstration and then run off – or like sometimes it was just really hard to get her back in there with the hands on. So sometimes I even had to have her verbally – I said can you – she’s like I’m sorry, I just can’t right now.” Almost all providers described feeling ill equipped to handle these situations, they also noted that they were likely to use child-directed intervention approaches with these families.

### Attitudes

There was a statistically significant association between providers’ perceived attitudes about coaching and their intentions to use caregiver coaching for all coaching components. Attitudes about coaching were also a primary theme in the interviews. Many providers openly described how their own attitudes influenced their use of caregiver coaching. Providers reported mixed feelings about caregiver coaching. Some providers described strong positive attitudes in favor of using a coaching model with caregivers. Statements such as “I hear people so resistant to this model and I just don’t understand it because it is so effective and so empowering. We know that parent coaching models are effective.” were commonly shared among providers who reported using caregiver coaching often during their EI sessions. However, other providers described challenges with caregiver coaching. One provider stated, “If you go too quickly with a parent who’s uncomfortable, they will freak out in a lot of different ways. They might stop the services. They might just not participate at all. There’s a lot of different ways they can react, and a lot of it’s just anxiety-based, because they’re not used to doing these kinds of things.” Providers with less favorable attitudes toward coaching also described the coaching model as not being responsive to some families’ needs. For example, one provider shared: “There’s just a lot of challenges in some of these homes that I don’t think is always considered when they’re talking about, delivery of (coaching) service and you know, how efficient it should be because it isn’t always like that.”

### Descriptive norms

The quantitative findings indicated that providers’ intentions to use some components of caregiver coaching were predicted by their perceptions of descriptive norms regarding other providers’ use of coaching strategies. Specifically, descriptive norms predicted providers’ intentions to use feedback and reflective problem-solving coaching strategies. Providers described perceptions that many other providers usually do not coach caregivers during sessions; instead, they use child-directed play-based intervention. Providers described inconsistency across providers related to the use of caregiver coaching, which presented challenges. One provider explained this: “I also had the instance where I’m the only one on the team coaching and the other therapists maybe are not coaching. The parent really wants me to come in and sit on the floor and play with their child. So, it’s a little harder at that point to have them buy in because the other therapists are basically playing and doing things with their child for an hour while they sit on the couch and drink their coffee.” This discussion regarding the lack of consistency across providers working in early intervention was pervasive across interviews and highlighted low descriptive norms for the use of caregiver coaching in early intervention. Providers also described a sense of isolation from their peers, which they believe contributed to the lack of consistency across providers. They described their daily routine involving traveling from one family’s home to the next, with little interaction or opportunities for collaboration with other EI providers. One provider stated: “I never go into the office. I never see anybody. I’m out in the field all by myself all the time. So, the opportunity for observing others or being trained and all that super minimal.”

### Injunctive norms

Our quantitative findings indicated that providers’ injunctive norms regarding some caregiver components were associated with their intentions to implement these practices. Our qualitative findings supported and expanded upon this finding. Specifically, providers discussed injunctive norms related to two groups that influenced their intentions to use caregiver coaching during early intervention sessions: caregivers and agency leaders. Providers’ perceptions of caregivers’ expectations were often described as a primary factor driving their decisions to use caregiver coaching. Providers often reported that they do not use caregiver coaching during EI sessions with families of young autistic children because caregivers do not want to be coached. Providers reported that caregivers prefer and expect child-directed intervention. A prominent theme was that caregivers expect them to deliver therapy to their child. One provider shared, “If I go into their house, they sometimes are like, oh, you’re the therapist, you work with my kid.” Other providers shared similar sentiments. Another stated, “Parents sometimes think that therapists are the experts. And so, we are the ones who are there to work.” These providers all indicated that they are less likely to use coaching with families who expect a child-directed intervention approach. Our qualitative findings also highlighted the potential role of agency leadership in shaping providers’ injunctive norms for caregiver coaching. For example, one provider said “[Agency leaders] really, really highlighted the importance of parent coaching and that the goal or the model of early intervention really is based on parent coaching and getting the family to be comfortable with these sessions.”

### Implementation leadership and climate

Importantly, the qualitative findings largely centered around individual-level factors, rather than organizational-level factors, that influenced the use of caregiver coaching in this context. There was relatively little mention of the role agency-level factors played in the use of the components of caregiver coaching. However, a few providers did discuss the importance of agency leadership or climate in qualitative interviews. Providers also mentioned specific training and support from their agency leaders as being instrumental to their use of caregiver coaching. For example, one provider shared: “My agency specifically, and I don’t know how ubiquitous this is, my agency specifically provides training on parent coaching, so I think that’s helpful just to have some – we tend to do a lot of playing at roles, so practicing what we would say in response to a parent refusing to do something or how we could coach for different, you know, specific things that we are noticing. Like we tend to do a lot of the role play type of things at my agency as a part of our training before we start [coaching].” These comments highlighted the role agency leaders and training played in providers’ views toward caregiver coaching.

## Discussion

Elucidating relationships among individual- and organizational-level determinants of implementation is a priority to advance implementation science [53], but few studies examine these factors together. The present study closely examined both individual- and organizational-level factors that can influence the use of a particular evidence-based practice, caregiver coaching, with a large sample of community-based providers. The findings from this evaluation offer insight into potential mechanisms underlying the implementation of EBPs in community settings. This study extends prior research that found that providers’ intentions to implement the components of caregiver coaching, and the individual-level factors that relate to intentions, vary across components [2]. The results of the present study suggest specific individual and organizational factors that implementation strategies could target and offer insight into how and why implementation strategies may work and the conditions under which specific implementation strategies may be most successful.

Consistent with [2], we found that the associations among intentions, psychological determinants of intentions, and organizational factors varied across core components of caregiver coaching. Importantly, this study used the same sample as [2] and extended that work by adding the organizational level variables into the model and including mixed methods. The individual-level relationships for intentions and determinants of intentions did not change when the organizational-level variables were added to the model, indicating a strong and persistent relationship among these constructs. This is also consistent with other findings from different settings and interventions, suggesting that providers’ intentions to use complex psychosocial interventions vary by intervention component [45, 54]. These results suggest that implementation strategies should target the individual components of a complex psychosocial intervention (e.g., providing caregivers feedback; engaging in reflection and problem solving), rather than the intervention package as a whole (e.g., “caregiver coaching”) to improve its implementation.

Qualitative results expanded on these findings by offering nuance in the constructs of interest and revealing an in-depth understanding of how providers describe the relationships between the key constructs in with each other. For example, providers expressed a wide range of attitudes about coaching caregivers; similarly, injunctive norms regarding caregivers’ expectations were particularly salient in the qualitative results. These results suggest that implementation strategies should include components that change provider and caregiver attitudes toward coaching as well as support providers in setting expectations for coaching with caregivers and in securing caregiver buy-in. Previous research has emphasized the importance of gaining stakeholder buy-in prior to implementing an innovation [55, 56]. Additionally, implementation strategies that help caregivers develop accurate expectations for early intervention, such as distributing educational information about the intervention or obtaining written commitments to participate in coaching [57] could reduce barriers to implementation. Little research has centered on the need to develop dyadic implementation strategies that concurrently and synergistically engage constituents from multiple constituent groups. Our findings indicate the need for dyadic implementation strategies targeting caregivers and providers simultaneously while focusing on different yet complimentary implementation levers. This approach warrants further evaluation as it has broad applications to implementation strategies which often are deployed in contexts with multiple levels of constituent groups.

Our quantitative results indicated that, with some exceptions, organizational-level climate and culture were not associated with most psychological factors. Implementation climate and implementation leadership consistently were associated with injunctive norms in the between-level models but were not associated with most other constructs of interest. Dimensions of general organizational culture and climate (as measured by the OSC) were not associated with determinants of intentions in most cases. It is possible that these findings are a function of the type of constructs measured, specifically global versus strategic organizational factors. Our findings indicated that global organizational factors were not associated with intentions; however, strategic organizational factors such as implementation climate and leadership were consistently associated with injunctive norms. Measuring strategic organizational variables likely added specificity and aided in identifying relationships among variables that were not evident in more global measures. Future research should more carefully evaluate the utility of measuring global versus strategic organizational variables in implementation research.

Although our quantitative results did show some relationships between strategic organizational factors and injunctive norms, our qualitative results largely centered around themes related to individual family and provider level constructs. This may be related to the fact that the early intervention providers in these service systems were mostly independent contractors, rather than salaried employees. They work independently, delivering services directly in the field with little opportunity for connection and collaboration among providers. Previous research has found that independent contractors are less likely to adopt EBPs and more likely to implement them with lower fidelity than salaried employees [57]. Organizational culture and climate may have played a smaller role in this sample than in other settings because contractors often have fewer opportunities to be influenced by the agency’s culture and climate. Implementation strategies focused on building connections and collaboration among providers, such as the use of learning collaboratives, building a coalition, or group facilitation [58], may be especially critical in these types of fragmented service systems. It also remains possible that organizational culture and climate are important in this context for outcomes that we did not measure here, such as provider burnout or turnover.

In some cases, the qualitative findings did not support the quantitative results. For example, the quantitative analyses did not find an association between providers’ self-efficacy and their intentions to use feedback with caregivers; however, providers often described feelings of discomfort delivering feedback to caregivers throughout the qualitative interviews. It is possible that providers’ feelings of discomfort with delivering feedback to caregivers did not reflect their sense of competence or self-efficacy with delivering feedback. For example, a provider could feel competent in delivering feedback to caregivers, but uncomfortable doing so.

Several study limitations are important to note. The early intervention providers reported on their intentions, determinants of intentions, and perspectives about caregiver coaching in community-based early intervention. They did not report on their actual behavior, and we do not have data regarding these providers’ actual use of caregiver coaching. Prior observational studies describe poor fidelity to caregiver coaching in community-based early intervention [6, 19], but these findings may not have applied to our sample. Including measures of providers’ use of caregiver coaching is an important direction for future research, as this will make it possible to test hypothesized relationships among organizational factors, intentions and implementation behavior (e.g., that environmental constraints may moderate the relationship between provider intentions and behavior; see Fig. 1). The practical significance of the observed quantitative relationships is also not clear. Third, there was considerable variability in the sample size of providers across agencies, although this is reflective of the variation of agency size in real-world community-based service settings. Additionally, the providers who participated in our qualitative interviews responded to our request for additional information, and were motivated to participate in our follow-up interviews. These providers may not represent the larger population of providers working in this field. Lastly, these data represent the associations between these constructs at a single point in time, rather than longitudinal or causal relationships. It is not possible to draw causal relationships from this type of cross-sectional and self-reported data. However, these findings offer important direction for future longitudinal research aimed at evaluating mechanisms and causal models in implementation science, as well as examining the practical significance of these relationships.

## Conclusion

The current study advances the literature by using mixed methods to examine theory-driven organizational-level and individual-level constructs. Results highlight the promise of tailored, multi-level implementation strategies that strategically target both organizational-level (e.g., leadership training on the importance of coaching to improve injunctive norms, communities of practice to improve descriptive norms) and individual-level constructs (e.g., education regarding the benefits of caregiver coaching to improve attitudes and targeted consultation regarding use of the individual coaching components to improve self-efficacy). Multifaceted implementation strategies that include strategies directed at each level are more likely to improve implementation in systems with barriers across levels. Strategies designed to be flexibly applied to meet the needs of individual providers are more cost-efficient and effective. This approach holds promise for improving the implementation of complex, multicomponent, psychosocial interventions in community-based service systems.

### Supplementary Information

**Additional file 1.**

**Additional file 1.**

## Data Availability

The datasets collected and analyzed during the current study are available from the corresponding author on reasonable request.
