# Supplementary material for: A mixed-methods evaluation of organization and individual factors influencing provider intentions to use caregiver coaching in community-based early intervention
Source: Implement Sci Commun. 2024 Feb 27;5:17. doi: 10.1186/s43058-024-00552-5 (PMC10900730; doi:10.1186/s43058-024-00552-5)
Supplement: Supplementary file 1 — Additional file 1. [file 43058_2024_552_MOESM1_ESM.docx]

**Supplemental Table 1**

*Descriptive statistics of OSC subscale scores*

|  | M | *SD* | Range |
| --- | --- | --- | --- |
| Proficiency culture T score (*N* = 18) | 57.61 | 5.00 | [48.61, 65.08] |
| Rigidity culture T score (*N* = 18) | 53.68 | 8.71 | [43.49, 70.82] |
| Resistance culture T score (*N* = 18) | 58.04 | 7.28 | [49.02, 71.38] |
| Engagement climate T Score (*N* = 18) | 62.74 | 5.43 | [46.82, 74.34] |
| Functionality climate T Score (*N* = 18) | 66.61 | 6.95 | [46.72, 74.34] |
| Stress climate T Score (*N* = 18) | 41.18 | 4.71 | [31.42, 52.47] |
