# Supplementary material for: A mixed-methods evaluation of organization and individual factors influencing provider intentions to use caregiver coaching in community-based early intervention
Source: Implement Sci Commun. 2024 Feb 27;5:17. doi: 10.1186/s43058-024-00552-5 (PMC10900730; doi:10.1186/s43058-024-00552-5)
Supplement: Supplementary file 2 — Additional file 1. [file 43058_2024_552_MOESM2_ESM.docx]

**Supplemental Table 2**

*Results from Multilevel Path Models with paths from Organizational Social Context (OSC) subscales to Psychological Determinants of Intentions and from Psychological Determinants to Intentions for the Five Parent Coaching Core Components (i.e., Feedback, Collaboration, Demonstration, Reflection/Problem Solving, Daily Routines). Between-level paths from Organizational Social Context (OSC) subscales to Psychological Determinants of Intentions are shown here.*

|  | Feedback | Collaboration | Demonstration | Reflection and Problem Solving | Daily Routines |
| --- | --- | --- | --- | --- | --- |
|  | Effect (*p*) | Effect (*p*) | Effect (*p*) | Effect (*p*) | Effect (*p*) |
| **Proficiency** |  |  |  |  |  |
| Proficiency →Self-efficacy | **.04 (.01)** | .02 (.06) | **.03 (.02)** | **.02 (.003)** | .03 (.07) |
| Proficiency →Attitudes | .03 (.74) | .01 (.81) | .03 (.20) | .04 (.56) | -.01 (.88) |
| Proficiency →Descriptive norms | .01 (.78) | .02 (.38) | .02 (.37) | .02 (.47) | -.001 (.99) |
| Proficiency →Injunctive norms | .02 (.66) | .002 (.94) | .01 (.41) | .02 (.56) | -.02 (.71) |
| **Rigidity** |  |  |  |  |  |
| Rigidity →Self-efficacy | .013 (.18) | .007 (.37) | .006 (.85) | .001 (.89) | -.001 (.87) |
| Rigidity →Attitudes | .055 (.09) | .028 (.42) | .018 (.39) | .019 (.76) | -.024 (.59) |
| Rigidity →Descriptive norms | .026 (.10) | .011 (.53) | .003 (.89) | .013 (.67) | -.003 (.92) |
| Rigidity →Injunctive norms | .018 (.38) | -.004 (.82) | .003 (.94) | .005 (.88) | -.02 (.41) |
| **Resistance** |  |  |  |  |  |
| Resistance →Self-efficacy | .005 (.72) | -.004 (.58) | -.002 (.81) | -.009 (.07) | -.013 (.051) |
| Resistance →Attitudes | .045 (.34) | -.008 (.84) | .005 (.80) | -.027 (.53) | -.061 (.11) |
| Resistance →Descriptive norms | .02 (.34) | -.011 (.58) | -.008 (.66) | -.007 (.74) | -.021 (.45) |
| Resistance →Injunctive norms | .012 (.65) | -.025 (.13) | -.002 (.90) | -.022 (.31) | -.038 (.09) |
| **Engagement** |  |  |  |  |  |
| Engagement →Self-efficacy | .03 (.98) | **.04 (.<.001)** | **.03 (.002**) | **.02 (.02)** | .02 (.28) |
| Engagement →Attitudes | .08 (.97 | .07 (.18) | .04 (.15) | .07 (.28) | .02 (.87) |
| Engagement →Descriptive norms | .02 (.99) | .02 (.79) | .02 (.17) | .03 (.46) | -.02 (.76) |
| Engagement →Injunctive norms | .02 (.99) | -.01 (.80) | .001 (.98) | .02 (.67) | -.03 (.44) |
| **Functionality** |  |  |  |  |  |
| Functionality →Self-efficacy | **.02 (.006)** | .01 (.50) | .01 (.12) | **.01 (.006)** | **.02 (.04)** |
| Functionality →Attitudes | .009 (.80) | .03 (.40) | .01 (.37) | .03 (.40) | .03 (.38) |
| Functionality →Descriptive norms | -.001 (.95) | .02 (.12) | -.002 (.85) | .02 (.37) | .02 (.39) |
| Functionality →Injunctive norms | .01 (.59) | .02 (.21) | .01 (.09) | .03 (.14) | .02 (.34) |
| **Stress** |  |  |  |  |  |
| Stress →Self-efficacy | .004 (.87) | -.01 (.40) | -.005 (.55) | -.006 (.32) | -.02 (.06) |
| Stress →Attitudes | .10 (.10) | .008 (.89) | .024 (.71) | -.001 (.99) | -.042 (.53) |
| Stress →Descriptive norms | .046 (.10) | -.012 (.68) | -.002 (.87) | -.01 (.76) | -.025 (.57) |
| Stress →Injunctive norms | .045 (.24) | -.02 (.49) | -.007 (.86) | -.012 (.77) | -.027 (.48) |

*Note*. Unstandardized coefficients are shown
